# Chloroplast genome data of *Luffa acutangula* and *Luffa aegyptiaca* and their phylogenetic relationships

**DOI:** 10.1016/j.dib.2020.106470

**Published:** 2020-10-28

**Authors:** Chutintorn Yundaeng, Wanapinun Nawae, Chaiwat Naktang, Jeremy R. Shearman, Chutima Sonthirod, Duangjai Sangsrakru, Thippawan Yoocha, Nukoon Jomchai, John R. Sheedy, Supat Mekiyanon, Methawat Tuntaisong, Wirulda Pootakham, Sithichoke Tangphatsornruang

**Affiliations:** a National Omics Center, National Science and Technology Development Agency, 111 Thailand Science Park, Paholyothin Road, Khlong Nueng, Khlong Luang, Pathum Thani, 12120, Thailand; b Chia Tai Company Limited, Phra Khanong District, Bangkok, Thailand

**Keywords:** *Luffa acutangula*, *Luffa aegyptiaca*, chloroplast genome, PacBio sequencing, comparative analysis

## Abstract

*Luffa acutangula* and *Luffa aegyptiaca* are domesticated plants in the family Cucurbitaceae. They are mainly cultivated in the tropical and subtropical regions of Asia. The chloroplast genomes of many Cucurbitaceae species were sequenced to examine gene content and evolution. However, the chloroplast genome sequences of *L. acutangula* and *L. aegyptiaca* have not been reported. We report the first complete sequences of *L. acutangula* and *L. aegyptiaca* chloroplast genomes obtained from Pacific Biosciences sequencing and use them to infer evolutionary relationships. The chloroplast genomes of *L. acutangula* and *L. aegyptiaca* are 157,202 and 157,275 bp, respectively. Both genomes possessed the typical quadripartite structure and contained 131 genes, including 87 coding genes, 36 tRNA genes and 8 rRNA genes. We identified simple sequence repeats (SSR) and single nucleotide polymorphisms (SNP) from both chloroplast genomes. Polycistronic mRNA was examined in *L. acutangula* and *L. aegyptiaca* using RNA sequences from Isoform sequencing to identify co-transcribed genes. IR size and locations were compared to other species and found to be relatively unchanged. Phylogenetic analysis confirmed the close relationship between *L. acutangula* and *L. aegyptiaca* in the Cucurbitaceae lineage and showed separation of the *Luffa* monophyletic clade from other species in the subtribe Sicyocae. The results obtained from this study can be useful for studying the evolution of Cucurbitaceae plants.

## Specifications Table

**Table:** 

Subject	Plant Science
Specific subject area	Genomic
Type of data	Tables Graph Figures Raw data Sequences
How data were acquired	Pacific Biosciences sequencing (PacBio RSII sequencing)
Data format	Chloroplast raw sequence data in FASTQ format Complete chloroplast genome sequence in FASTA format
Parameters for data collection	Genomic DNA was extracted from fresh leaves of *L. acutangula* and *L. aegyptiaca* plants to derive from Chia Tai Company Limited. Leaves of 61 accessions of *L. acutangula* and 23 accessions of *L. aegyptiaca* seedlings (Chia Tai Co, Ltd) were harvested and genomic DNA isolated.
Description of data collection	PacBio libraries were prepared to sequence on the PacBio RSII sequencing for complete chloroplast genomes assembly. Illumina Hiseq X ten libraries with 150 bp pair-end were constructed and sequenced for simple sequence repeats (SSR) and single nucleotide polymorphism (SNP) identifications.
Data source location	Institution: National Science and Technology Development Agency, Region: Khlong Luang, Pathum Thani Country: Thailand
Data accessibility	All data in this article are available at NCBI, BioProject number PRJNA639390. Chloroplast raw sequence data with this article are accessible under SRA accession number SRR12011300 (*L. acutangula*) and SRR12011301 (*L. aegyptiaca*). Direct URL to data: https://www.ncbi.nlm.nih.gov/sra/?term=SRR12011300 https://www.ncbi.nlm.nih.gov/sra/?term=SRR12011301 Complete chloroplast sequence data are accessible at NCBI under GenBank accession number MT381996 (*L. acutangula*) and MT381997 (*L. aegyptiaca*). Direct URL to data: https://www.ncbi.nlm.nih.gov/genome/?term=MT381996 https://www.ncbi.nlm.nih.gov/genome/?term=MT381997 Isoform sequencing (Iso-seq) data of *L. acutangula* [SRA accession number: SRR11445640] and *L. aegyptiaca* [SRA accession number: SRR11452010] were obtained from NCBI [1]. Direct URL to data: https://www.ncbi.nlm.nih.gov/sra/?term=SRR11445640 https://www.ncbi.nlm.nih.gov/sra/?term=SRR11452010

## Value of the Data

• *L. acutangula* and *L. aegyptiaca* chloroplast genomes are sources of molecular data that confirm complex evolutionary relationships and support the need for phylogenetic research in various plant groups.
• The complete chloroplast genome data could be utilized in the genetics, biotechnology, plant breeding, and ecology fields.
• The sequence variation among the chloroplast genomes of *Luffa* sp. and other representatives of the family Cucurbitaceae enhances the understanding of their phylogenetic relationships.
• Polymorphisms in the chloroplast genome (e.g., simple sequence repeats (SSRs) or single nucleotide polymorphisms (SNPs)) can be used to develop potential molecular markers and study evolutionary patterns of *Luffa* sp. and closely related species.

## 1 Data Description

**Fig. 1:**
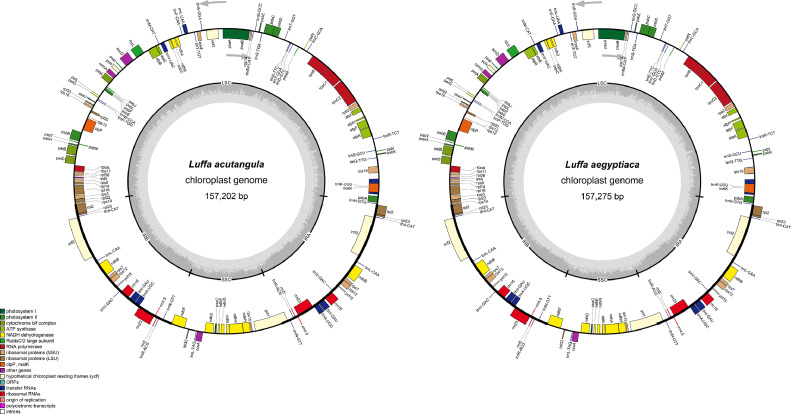
The chloroplast genomes of *L. acutangula* and *L. aegyptiaca*. Genes shown outside of the circle are transcribed counterclockwise, while those inside are transcribed clockwise, as shown by the arrows. The functions of genes are grouped by color. Asterisks indicate intron-containing genes.

**Fig. 2:**
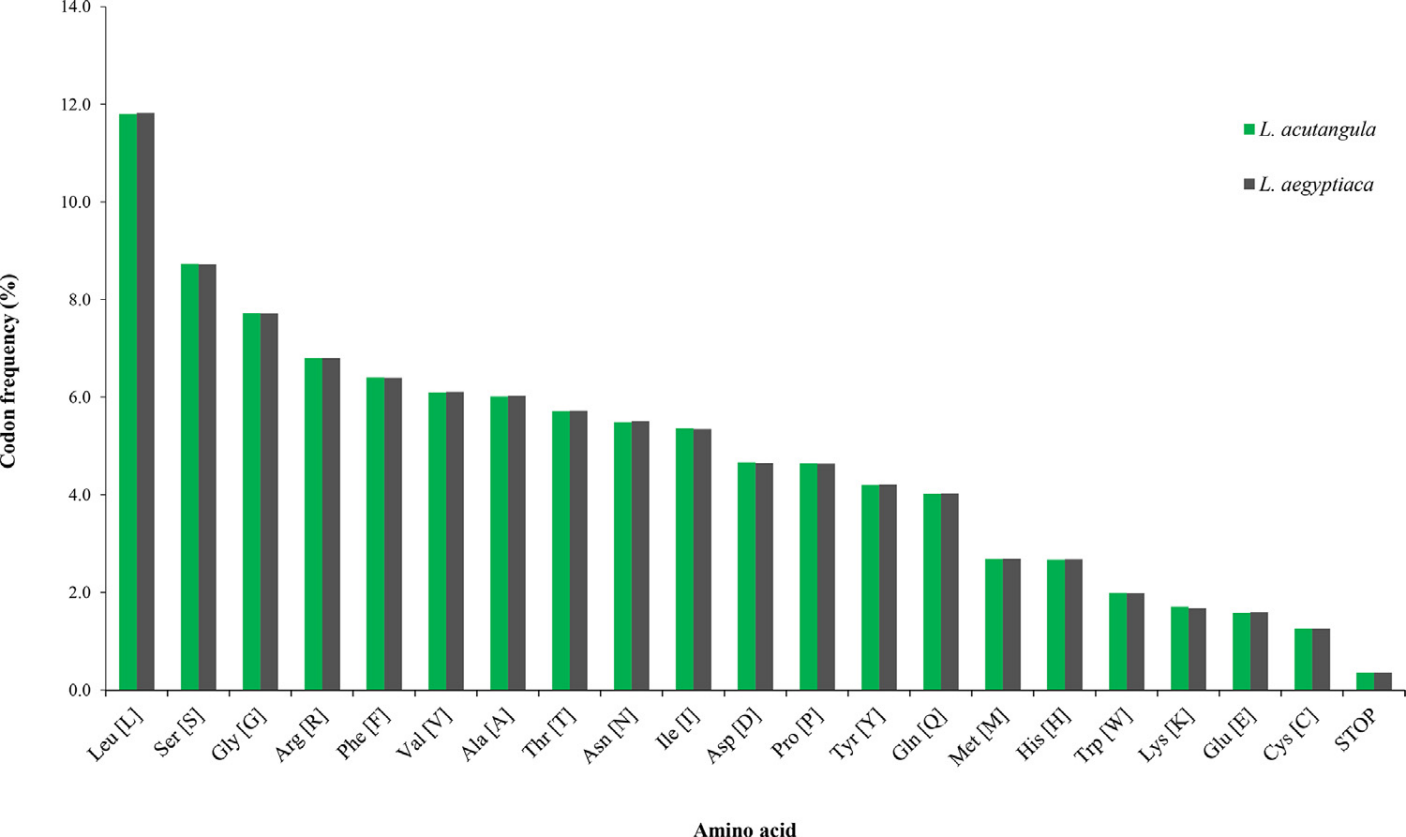
Amino acid frequencies in *L. acutangula* and *L. aegyptiaca* protein-coding sequences.

**Fig. 3:**
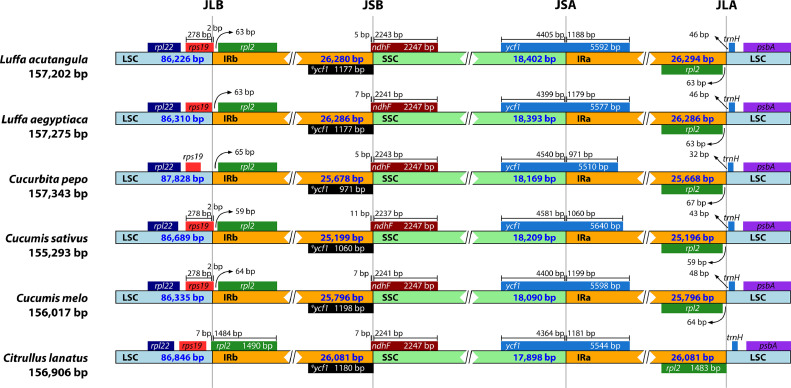
Comparison of the chloroplast genome borders of the LSC, SSC, and IR regions among six species, ψ partial fragment of the *ycf1* gene.

**Fig. 4:**
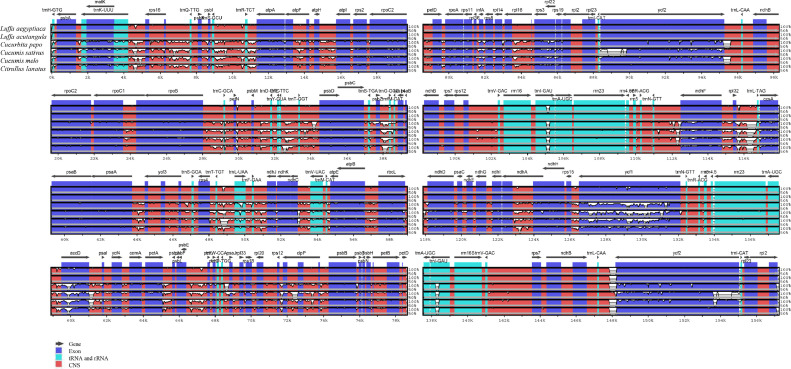
Alignment of chloroplast genome sequences, showing percent similarity, among six species using *L. acutangula* as a reference.

**Fig. 5:**
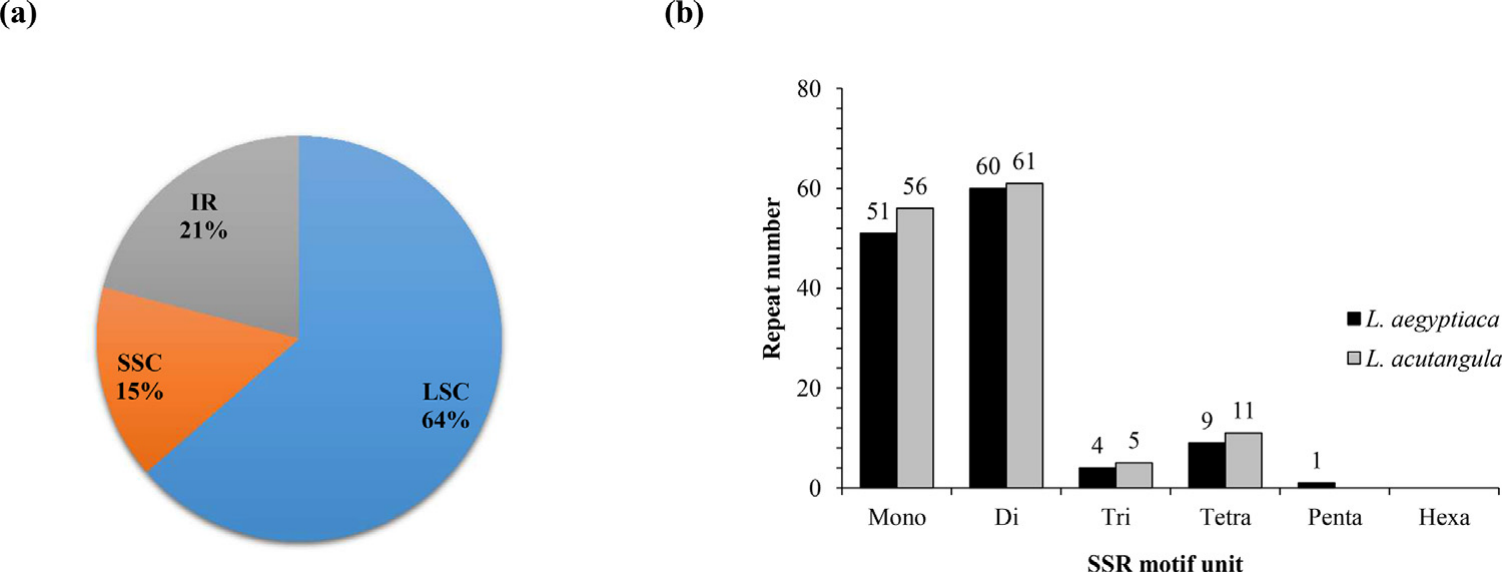
Simple sequence repeat (SSR) analysis in *L. acutagula* and*L. aegyptiaca* chloroplast genomes. (a) SSR percentage in the LSC, SSC and IR regions, (b) Number of SSR per motif size.

**Fig. 6:**
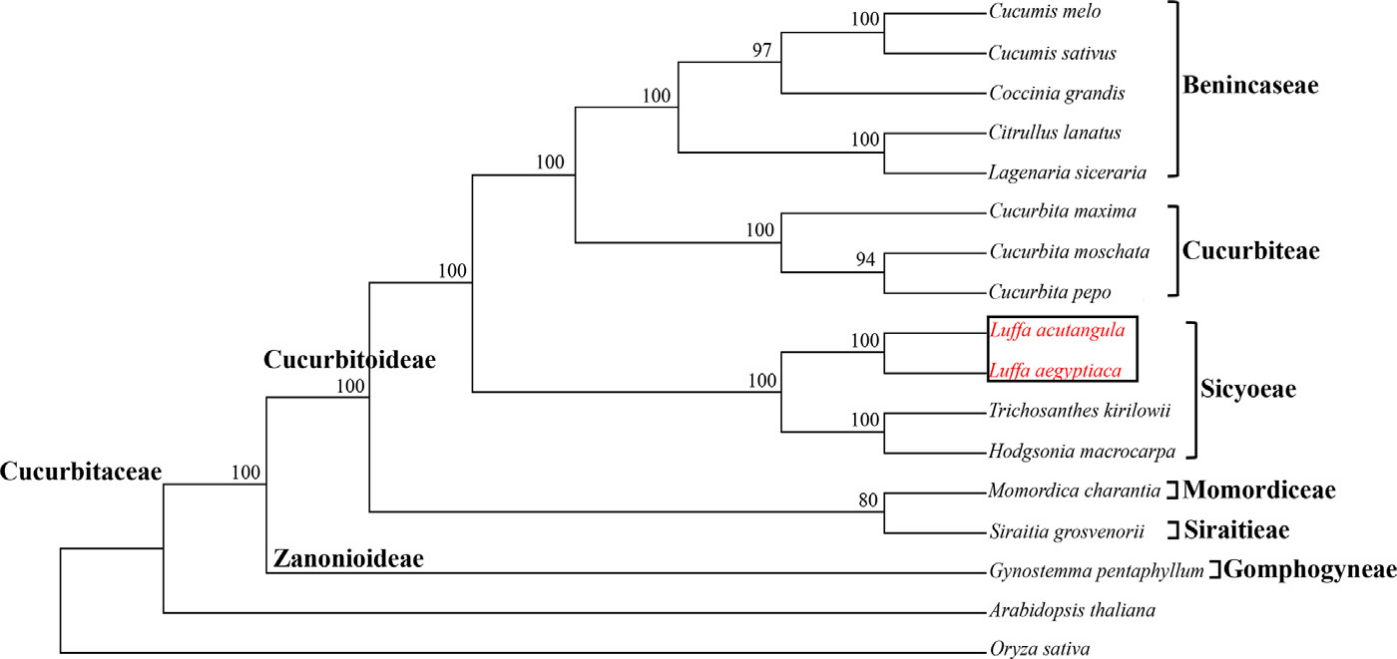
Phylogenetic relationship of 17 species within Cucurbitaceae family based on 66 protein-coding chloroplast genes. *O. sativa* and *A. thaliana* are outgroups. Numbers above the node are the bootstrap values of maximum likelihood (ML) analysis.

The complete chloroplast genomes of *L. acutangula* and *L. aegyptiaca* were assembled using long read sequences obtained from PacBio sequencing and annotated for gene content. The chloroplast genome sequences and annotated genes are available through NCBI accession number MT381996 (*L. acutangula*) and MT381997 (*L. aegyptiaca*). Both chloroplast genomes had the typical quadripartite structure, which consists of a small single-copy region (SSC) and a large single-copy region (LSC), separated by a pair of inverted repeats (IRs) (Fig. 1, Table 1). Both chloroplast genomes encoded 131 genes, including 87 protein-coding genes, 36 tRNA genes and 8 rRNA genes (Table 2, Table 3). The codon-usage frequencies were calculated for the protein-coding genes and tRNA genes of the *L. acutangula* and *L. aegyptiaca* chloroplast genomes (Fig 2, Table 4). Length and position of the LSC and SSC regions and genetic variation the chloroplast genomes were examined among *L. acutangula, L. aegyptiaca* and other species in the family Cucurbitaceae (Fig. 3 and 4). Simple sequence repeats (SSR) (Fig. 5, supplementary Table S1), single nucleotide polymorphisms (SNP) (Table 5) and RNA editing events (Table 6) in both*L. acutangula* and *L. aegyptiaca* chloroplast genomes were identified. Polycistronic transcript sequences were similar in *L. acutangula* and *L. aegyptiaca* chloroplast genomes (Table 7, supplementary Table S2). Furthermore, a phylogenetic analysis of *Luffa* and several Cucurbitaceae species placed *L. acutangula* and *L. aegyptiaca* closely related to Tricosanthes and Hodgsonia in the Sicyoeae tribe (Fig. 6).

**Table 1:** Chloroplast genome features among Cucurbitaceae species.

	*L. acutangula*	*L. aegyptiaca*	*C. lanatus*	*C. melo*	*C. sativus*	*C. pepo*
Genome size (bp)	157,202	157,275	156,906	156,017	155,293	157,343
LSC size (bp)	86,226	86,310	86,846	86,335	86,689	87,828
SSC size (bp)	18,402	18,393	17,898	18,090	18,209	18,169
IRs size (bp)	26,280	26,286	26,081	25,796	25,199	25,678
GC content (%)	37.14	37.12	37.18	36.92	37.08	37.16
LSC GC content (%)	34.96	34.93	34.94	34.67	34.85	34.91
SSC GC content (%)	31.02	31.04	31.54	30.94	31.83	31.44
IRs GC content (%)	42.86	42.86	42.84	42.79	42.83	43.05
No. of genes	131	131	124	135	133	131
No. of CDS	87	87	87	90	89	86
No. of tRNA	36	36	29	37	37	37
No. of rRNA	8	8	8	8	8	8
No. of CDS with intron	15	15	10	16	15	15
Gene coding density (%)	50.08	50.04	49.74	51.74	50.06	46.60
Genbank accession number	MT381996	MT381997	NC_032008	NC_015983	NC_007144	NC_038229

**Table 2:** List of genes present in *L. acutangula* and *L. aegyptiaca* chloroplast genomes.

Category	Gene groups	Gene name
Photosynthesis	Photosystem I (5)	*psaA, psaB, psaC, psaI, psaJ*
	Photosystem II (15)	*psbA, psbB, psbC, psbD, psbE, psbF, psbH, psbI, psbJ, psbK, psbL, psbM, psbN, psbT, psbZ*
	Cytochome b6/f complex (6)	petA, petB*, petD*, *petG, petL, petN*
	ATP synthase (6)	atpA, atpB, atpE, atpF*, *atpH, atpI*
	Rubisco large subunit (1)	*rbcl*
	NADH dehydrogenase (12)	ndhA*, *ndhB* (× 2)*, *ndhC, ndhD, ndhE, ndhF, ndhG, ndhH, ndhI, ndhJ, ndhK*
Self-replication	Large subunit Ribosomal protein (11)	*rpl2* (× 2)*, rpl14, rpl16*, *rpl20, rpl22, rpl23* (× 2), *rpl32, rpl33, rpl36*
	Small subunit ribosomal protein (14)	*rps2, rps3, rps4, rps7* (× 2), *rps8, rps11, rps12* (× 2)*, rps14, rps15, rps16*, *rps18, rps19*
	RNA polymerase (4)	rpoA, rpoB, rpoC1*, *rpoC2*
	Ribosomal RNAs (8)	*rrn4.5* (× 2),*rrn5* (× 2), *rrn16* (× 2), *rrn23* (× 2)
	Transfer RNAs (36)	*trnA-UGC* (× 2)*, *trnC-GCA, trnD-GTC, trnE-TTC, trnF-GAA, trnfM-CAT, trnG-GCC, trnH-GTG, trnI-CAT* (× 2), *trnI-GAU* (× 2)*, trnK-UUU*, *trnL-CAA* (× 2), trnL-TAG, trnL-UAA*, *trnM-CAT, trnN-GTT* (× 2), *trnP-TGG, trnQ-TTG, trnR-ACG* (× 2), *trnR-TCT, trnS-GCU, trnS-GGA, trnS-TGA, trnT-GGT, trnT-TGT, trnV-GAC* (× 2), trnV-UAC*, *trnW-CCA, trnY-GUA*
Other genes	Acetyl-CoA carboxylase gene (1)	*accD*
	c-type cytochrome biogenesis (1)	*ccsA*
	ATP-dependent protease subunit (1)	clpP*
	Maturease (1)	*matK*
	Membrane protein (1)	*cemA*
	Proteins of unknown function (7)	*ycf1, ycf2* (× 2), ycf3*, *ycf4, ycf15* (× 2)
	Translation-related gene (1)	*infA*

⁎ Gene with intron(s)

**Table 3:** Genes with intron(s) in*L. acutangula* and *L. aegyptiaca* chloroplast genomes.

Gene	Location	Species									
		*L. acutangula*					*L. aegyptiaca*				
		Exon I	Intron I	Exon II	Intron II	Exon III	Exon I	Intron I	Exon II	Intron II	Exon III
		(bp)	(bp)	(bp)	(bp)	(bp)	(bp)	(bp)	(bp)	(bp)	(bp)
*rps16*	LSC	42	855	213	-	-	45	856	213	-	-
*atpF*	LSC	144	755	411	-	-	144	757	411	-	-
*rpoC1*	LSC	432	753	1611	-	-	432	756	1611	-	-
*ycf3*	LSC	126	740	228	743	153	126	740	228	740	156
*clpP*	LSC	69	847	288	613	228	69	835	297	615	225
*petB*	LSC	6	783	642	-	-	9	780	642	-	-
*petD*	LSC	9	727	474	-	-	9	732	474	-	-
*rpl16*	LSC	9	1100	402	-	-	9	1098	402	-	-
*rpl2*	IRb	390	665	435	-	-	390	665	435	-	-
*ndhB*	IRb	777	686	756	-	-	777	686	756	-	-
*rps12*	IRb	114	28918	234	537	27	114	28346	234	537	27
*ndhA*	SSC	552	1155	540	-	-	552	1146	540	-	-
*rps12*	IRa	114	71157	234	537	27	114	71136	234	537	27
*ndhB*	IRa	786	677	756	-	-	777	686	756	-	-
*rpl2*	IRa	390	665	435	-	-	393	662	435	-	-

**Table 4:** The codon-anticodon recognition pattern and codon usage for*L. acutangula* and *L. aegyptiaca* chloroplast genomes.

Amino acid	Codon	Frequency^a^		RSCU		trn^b^
		*L. acutangula*	*L. aegyptiaca*	*L. acutangula*	*L. aegyptiaca*	
Phe	UUU	957	957	1.29	1.29	*trnF-GAA*
Phe	UUC	530	529	0.71	0.71	
Leu	UUA	860	860	1.88	1.88	*trnL-UAA*
Leu	UUG	556	556	1.22	1.22	*trnL-CAA*
Leu	CUU	585	585	1.28	1.28	*trnL-TAG*
Leu	CUC	190	189	0.42	0.41	
Leu	CUA	377	379	0.82	0.83	
Leu	CUG	174	176	0.38	0.38	
Ile	AUU	84	83	1.45	1.45	*trnI-GAU*
Ile	AUC	474	472	0.63	0.63	
Ile	AUA	688	687	0.92	0.92	*trnI-CAT*
Met	AUG	624	625	1	1	*trnM-CAT*
						*trnfM-CAT*
Val	GUU	508	507	1.43	1.43	*trnV-GAC*
Val	GUC	181	183	0.51	0.52	
Val	GUA	530	531	1.5	1.5	*trnV-UAC*
Val	GUG	198	198	0.56	0.56	
Ser	UCU	571	566	1.69	1.68	*trnS-GGA*
Ser	UCC	319	322	0.94	0.95	
Ser	UCA	428	429	1.27	1.27	*trnS-UGA*
Ser	UCG	189	188	0.56	0.56	
Pro	CCU	413	410	1.53	1.52	*trnP-UGG*
Pro	CCC	201	203	0.75	0.75	
Pro	CCA	315	314	1.17	1.17	
Pro	CCG	150	151	0.56	0.56	
Thr	ACU	534	535	1.61	1.61	*trnT-GGU*
Thr	ACC	248	248	0.75	0.75	
Thr	ACA	397	399	1.2	1.2	*trnT-UGU*
Thr	ACG	149	147	0.45	0.44	
						
Ala	GCU	634	635	1.81	1.81	*trnA-UGC*
Ala	GCC	231	232	0.66	0.66	
Ala	GCA	384	383	1.1	1.09	
Ala	GCG	149	150	0.43	0.43	
Tyr	UAU	782	784	1.6	1.6	*trnY-GUA*
Tyr	UAC	194	194	0.4	0.4	
STOP	UAA	54	54	1.93	1.93	
STOP	UAG	16	16	0.57	0.57	
His	CAU	475	477	1.53	1.53	*trnH-GTG*
His	CAC	147	146	0.47	0.47	
Gln	CAA	719	720	1.54	1.54	*trnQ-TTG*
Gln	CAG	215	216	0.46	0.46	
Asn	AAU	983	982	1.54	1.53	*trnN-GTT*
Asn	AAC	293	298	0.46	0.47	
Lys	AAA	48	42	1.5	1.5	*trnK-UUU*
Lys	AAG	350	348	0.5	0.5	
Asp	GAU	873	871	1.61	1.61	*trnD-GTC*
Asp	GAC	211	209	0.39	0.39	
Glu	GAA	20	22	1.49	1.49	*trnE-TTC*
Glu	GAG	348	349	0.51	0.51	
Cys	UGU	216	216	1.47	1.47	*trnC-GCA*
Cys	UGC	78	78	0.53	0.53	
STOP	UGA	14	14	0.5	0.5	
Trp	UGG	464	462	1	1	*trnW-CCA*
Arg	CGU	354	354	1.34	1.34	*trnR-ACG*
Arg	CGC	103	100	0.39	0.38	*trnR-TCT*
Arg	CGA	368	370	1.4	1.41	
Arg	CGG	113	112	0.43	0.43	
Ser	AGU	401	399	1.19	1.18	*trnS-GCU*
Ser	AGC	121	122	0.36	0.36	
Arg	AGA	474	478	1.8	1.82	
Arg	AGG	168	166	0.64	0.63	
Gly	GGU	606	606	1.35	1.35	*trnG-GCC*
Gly	GGC	166	167	0.37	0.37	
Gly	GGA	727	727	1.62	1.62	
Gly	GGG	295	292	0.66	0.65	

*RSCU (Relative synonymous codon usage) value ≥ 1.00

a Frequency of codon usage in 23,224 and 23,220 codons in all potential protein-coding genes of *L. acutangula* and *L. aegyptiaca*, respectively;

b Gene encoding transfer RNA

**Table 5:** Candidate single nucleotide polymorphisms (SNPs) identified in CDS between the reference (*L. Acutangula*) and *L. aegyptiaca*.

Position	Reference	L. aeg	Sustitutionsa	Gene	Function
1973	T	C	NS	*matK*	Maturease K
3132	G	T	S	*matK*	Maturease K
5299	T	G	NS	*rps16*	30S ribosomal protein S16
8127	C	A	NS	*psbK*	Photosystem II reaction center protein K
8217	C	A	NS	*psbK*	Photosystem II reaction center protein K
12059	G	T	S	*atpA*	ATP synthase subunit alpha
13328	G	T	S	*atpF*	ATP synthase subunit b
17060	G	T	S	*rps2*	30S ribosomal protein S2
17982	C	A	NS	*rpoC2*	DNA-directed RNA polymerase subunit beta
18665	C	A	NS	*rpoC2*	DNA-directed RNA polymerase subunit beta
19148	C	T	S	*rpoC2*	DNA-directed RNA polymerase subunit beta
19540	C	A	NS	*rpoC2*	DNA-directed RNA polymerase subunit beta
20274	G	T	NS	*rpoC2*	DNA-directed RNA polymerase subunit beta
20678	A	G	S	*rpoC2*	DNA-directed RNA polymerase subunit beta
20777	A	G	S	*rpoC2*	DNA-directed RNA polymerase subunit beta
25097	G	T	S	*rpoB*	DNA-directed RNA polymerase subunit beta
26705	C	T	S	*rpoB*	DNA-directed RNA polymerase subunit beta
27002	C	T	S	*rpoB*	DNA-directed RNA polymerase subunit beta
35125	G	C	NS	*psbD*	Photosystem II D2 protein
51601	G	T	NS	*ndhJ*	NAD(P)H-quinone oxidoreductase subunit J
52335	G	T	S	*ndhK*	NAD(P)H-quinone oxidoreductase subunit K
55091	A	T	S	*atpE*	ATP synthase epsilon chain
55260	T	G	NS	*atpB*	ATP synthase subunit beta
55588	C	A	S	*atpB*	ATP synthase subunit beta
56576	G	A	NS	*atpB*	ATP synthase subunit beta
57691	T	G	NS	*rbcL*	Ribulose bisphosphate carboxylase large chain
59684	A	C	NS	*accD*	Acetyl-coenzyme A carboxylase carboxyl transferase subunit beta
59876	C	A	NS	*accD*	Acetyl-coenzyme A carboxylase carboxyl transferase subunit beta
59878	C	G	NS	*accD*	Acetyl-coenzyme A carboxylase carboxyl transferase subunit beta
59913	G	C	S	*accD*	Acetyl-coenzyme A carboxylase carboxyl transferase subunit beta
60037	A	G	S	*accD*	Acetyl-coenzyme A carboxylase carboxyl transferase subunit beta
60042	T	G	S	*accD*	Acetyl-coenzyme A carboxylase carboxyl transferase subunit beta
60169	T	C	NS	*accD*	Acetyl-coenzyme A carboxylase carboxyl transferase subunit beta
60287	C	A	S	*accD*	Acetyl-coenzyme A carboxylase carboxyl transferase subunit beta
60384	G	C	S	*accD*	Acetyl-coenzyme A carboxylase carboxyl transferase subunit beta
60417	C	G	S	*accD*	Acetyl-coenzyme A carboxylase carboxyl transferase subunit beta
60615	C	G	S	*accD*	Acetyl-coenzyme A carboxylase carboxyl transferase subunit beta
60665	G	T	S	*accD*	Acetyl-coenzyme A carboxylase carboxyl transferase subunit beta
60914	G	C	NS	*accD*	Acetyl-coenzyme A carboxylase carboxyl transferase subunit beta
60921	T	G	S	*accD*	Acetyl-coenzyme A carboxylase carboxyl transferase subunit beta
60963	A	G	S	*accD*	Acetyl-coenzyme A carboxylase carboxyl transferase subunit beta
62698	C	A	S	*ycf4*	Proteins of unknown function
63405	C	A	S	*cemA*	Chloroplast envelope membrane protein
63691	A	C	NS	*cemA*	Chloroplast envelope membrane protein
64793	G	A	S	*petA*	Cytochrome f
67969	T	G	S	*petG*	Cytochrome b6-f complex subunit 5
112795	T	G	NS	*ndhF*	NAD(P)H-quinone oxidoreductase subunit 5
112868	C	G	NS	*ndhF*	NAD(P)H-quinone oxidoreductase subunit 5
112869	C	A	NS	*ndhF*	NAD(P)H-quinone oxidoreductase subunit 5
113666	C	A	S	*ndhF*	NAD(P)H-quinone oxidoreductase subunit 5
114616	C	G	NS	*ndhF*	NAD(P)H-quinone oxidoreductase subunit 5
114678	G	A	NS	*ndhF*	NAD(P)H-quinone oxidoreductase subunit 5
117774	T	C	S	*ccsA*	Cytochrome c biogenesis protein

Note: L. aeg, *Luffa aegyptiaca*; a Ns: Non-synonymous, S: Synonymous

**Table 6:** Comparison of RNA editing patterns in *L. acutangula* and *L. aegyptiaca* chloroplast genomes with other species.

Location	Gene	AA position	Codon conversion	AA Change	Substitution	L. acutangula	L. aegyptiaca	C. sativus	C. pepo	A. thaliana	N. tabacum
LSC	*atpA*	258	uCa→uUa	S→L	Nonsynonymous	(-)	(+)	(-)	(-)	(-)	(-)
		305	uCa→uUa	S→L	Nonsynonymous	(-)	(+)	(-)	(-)	(-)	(-)
		383	uCa→uUa	S→L	Nonsynonymous	(-)	(+)	(-)	(-)	(-)	(-)
	*atpF*	31	cCa→cUa	P→L	Nonsynonymous	(+)	(+)	(+)	(+)	(+)	(+)
	*rps2*	83	uCa→uUa	S→L	Nonsynonymous	(-)	(+)	(+)	(+)	-	(+)
	*rpoC2*	1,245	uCa→uUa	S→L	Nonsynonymous	(+)	(+)	(+)	(+)	-	(+)
	*rpoB*	809	uCa→uUa	S→L	Nonsynonymous	(-)	(+)	(+)	(+)	(+)	(+)
	*ndhK*	22	uCa→uUa	S→L	Nonsynonymous	(+)	(+)	(-)	(-)	(-)	(-)
	*petA*	273	Cag→Uag	Q→Q	Synonymous	(-)	-	(-)	(-)	(-)	(-)
		276	gCg→gUg	A→S	Nonsynonymous	(-)	(+)	(-)	(-)	(-)	(-)
		279	guC→guU	V→V	Synonymous	(-)	-	(-)	(-)	(-)	(-)
	*psbJ*	20	cCu→cUu	P→L	Nonsynonymous	(+)	(+)	(-)	(-)	(-)	(-)
	*psbF*	26	uCu→uUu	S→F	Nonsynonymous	(+)	(+)	(+)	(-)	(+)	(+)
	*rpoA*	67	uCu→uUu	S→F	Nonsynonymous	(+)	(+)	(-)	(-)	(-)	(-)
		277	uCa→uUa	S→L	Nonsynonymous	(+)	(+)	(+)	(+)	-	(+)
	*rps11*	36	uuC→uuU	F→F	Synonymous	-	-	(-)	(-)	(-)	(-)
IRb	*rpl23*	24	uCu→uUu	S→F	Nonsynonymous	(-)	(+)	(-)	(-)	(-)	(-)
SSC	*ndhD*	97	uCa→uUa	S→L	Nonsynonymous	(+)	(-)	(-)	(-)	(-)	(-)
		194	uCa→uUa	S→L	Nonsynonymous	(+)	(+)	(-)	(-)	(-)	(-)
		262	uCa→uUa	S→L	Nonsynonymous	(-)	(+)	(-)	(-)	(-)	(-)
		265	uCg→uUg	S→L	Nonsynonymous	(+)	(-)	(-)	(-)	(-)	(-)
	*ndhE*	77	cCa→cUa	P→L	Nonsynonymous	(+)	(+)	(-)	(-)	(-)	(-)
	*ndhA*	114	uCa→uUa	S→L	Nonsynonymous	(+)	(+)	(-)	(-)	(+)	(+)
	*ndhH*	169	Cau→Uau	H→Y	Nonsynonymous	(+)	(+)	(-)	(-)	(-)	(-)

Capital letters in codon triplets indicate target nucleotides; AA, Amino acid; (+), editing; (-), no editing; -, U encoded in the DNA (no editing); Blank space, Silent mutation

**Table 7:** Polycistronic gene clusters in *L. acutangula* and *L. aegyptiaca* chloroplast genomes.

Function	Gene cluster	Luffa acutangula			Luffa aegyptiaca		
		Genes	Position	Length (bp)	Genes	Position	Length (bp)
ATP synthase	atp-1	*atpI+atpH*	16,507..14,566	1,942	atpI+atpH	16,511..14,570	1,942
Ribosomal protein, ATP synthase	atp-2	*rps2+atpI+atpH*	17,422..14,566	2,857	rps2+atpI	17,432..15,768	1,665
NADH oxidoreductase	ndh-1	*ndhC+ndhK+ndhJ*	52,894..51,215	1,680	ndhC+ndhK+ndhJ	52,970..51,292	1,679
NADH oxidoreductase	ndh-2	*ndhE+psaC+ndhD*	120,578..118,128	2,451	ndhE+psaC+ndhD	120,668..118,224	2,445
Photosystem II	psb-1	*psbE+psbF+psbL+psbJ*	66,388..65,615	774	psbE+psbF+psbL+psbJ	66,493..65,721	773
Ribosomal protein	rpl-1	*rpl14+rps8+infA+* *rpl36+rps11*	82,936..80,856	2,081	rpl16+rpl14+rps8+ infA+rpl36+rps11	84,678..80,945	3,734
Ribosomal protein	rpl-2	-	-	-	rpl22+rps3	85,963..84,819	1,145
Ribosomal protein	rpl-3	-	-	-	rpl23+rpl2+rps19	88,163..86,033	2,131
Ribosomal protein	rps-1	-	-	-	rps12+rpl20	71,652..70,393	1,260
Ribosomal protein	rps-2	-	-	-	rps19+rpl22+rps3	86,311..84,819	1,493
Ribosomal protein, NADH oxidoreductase	rps-3	*rps15+ndhH*	126,075..124,517	1,559	rps15+ndhH	126,156..124,599	1,558
Ribosomal RNAs	rrn-1	*rrn23+rrn4.5+rrn5*	106,587.109,977	3,391	rrn23+rrn4.5+rrn5	106,675..110,065	3,391

## 2 Experimental Design, Materials and Methods

### 2.1 DNA extraction, sequencing and assembly

Young leaves of *L. acutangula* (ridge gourd) and *L. aegyptiaca* (smooth gourd) plants from Chia Tai Company Limited were collected at National Omics Center, Thailand Science Park, Pathum Thani, Thailand in March 2019 for DNA extraction. Genomic DNA was extracted using a CTAB method [2]. Total DNA was examined using a NanoDrop One spectrophotometer (Thermo Scientific, Wilmington, USA) and visualized by pulsed-field gel electrophoresis (PFGE). High quality DNA was used to construct PacBio libraries according to the ‘Procedure & Checklist—20 Kb Template Preparation Using Bluepippin Size Selection System’ protocol and sequenced on the PacBio RSII system. The short PacBio reads were used to correct the long PacBio reads and the corrected long reads were assembled using CANU version 1.4 software [3]. The resulting contigs were blasted against the plastid genome database to identify any chloroplast contigs, which were used to construct full chloroplast genomes.

Young leaves of *L. acutangula* and *L. aegyptiaca* seedlings (Chia Tai Co, Ltd) were harvested and genomic DNA isolated using the High Pure PCR Template Preparation kit of Roche. Genomic DNA was examined using a NanoDrop One spectrophotometer (Thermo Scientific, Wilmington, USA). High quality DNA was used to prepare Illumina Hiseq X Ten libraries and 150 bp pair-end sequencing was performed by Novogene, Singapore according to standard Illumina protocols.

### 2.2 Chloroplast genome annotation

The assembled chloroplast genomes of *L. acutangula* and *L. aegyptiaca* were annotated using GeSeq MPI-MP CHLOROBOX tool [4], specifically HMMER, tRNAscan and ARAGORN. An annotated genome map was generated using Organellar Genome DRAW (OGDRAW) [5]. Finally, the preliminary annotations were corrected manually to ensure that the correct start and stop positions were reported.

### 2.3 Codon usage analysis

*L. acutangula* and *L. aegyptiaca* coding sequences were used to calculate relative synonymous codon usage (RSCU) value using CodonW version 1.4.2 software [6]. Codon usage frequency was calculated and expressed as the number of codons encoding the same amino acid divided by the total number of codons [7].

### 2.4 Comparative structure analysis

IR regions in the chloroplast genomes of *L. acutangula, L. aegyptiaca, Cucumis melo* (NC_015983), *Cucumis sativus* (NC_007144), *Citrullus lanatus* (NC_032008), and *Cucurbita pepo* (NC_038229) were compared using IRscope software [8]. Sequences of all analyzed chloroplast genomes were aligned using LAGAN mode of mVISTA alignment software [9] (http://genome.lbl.gov/vista/mvista/submit.shtml).

### 2.5 Simple sequence repeat (SSR) analysis

*L. acutangula* and *L. aegyptiaca* chloroplast genomes were scanned for simple sequence repeats (SSRs) using MIcroSAtellite (MISA) identification tool [10]. The length threshold of minimum repetitive units were set to ten repeats for mono-nucleotide repeats, four repeats for di- and tri-nucleotide repeats, and three repeats for tetra-, penta- and hexa-nucleotide repeats according to the method of Ivanova and co-workers [11].

### 2.6 Single nucleotide polymorphism (SNP) identification

Illumina sequences were mapped to the chloroplast genomes using Burrows-Wheeler Aligner (BWA-MEM) software [12]. SNPs were identified from *L. acutangula* and *L. aegyptiaca* using Genome Analysis Toolkit (GATK) software v 4.1.2.0 [13]. All SNPs were filtered with criteria of read depth ≥ 20 and missing data ≤ 10%.

### 2.7 RNA editing analysis and polycistronic mRNA in chloroplast genomes

RNA sequencing of *L. acutangula* [SRA accession number: SRR11445640] and *L. aegyptiaca* [SRA accession number: SRR11452010] from isoform sequencing (Iso-seq) were obtained from a previous study of Pootakham et al. (2020) [1]. These long-read sequences were mapped to their corresponding chloroplast genomes using BWA-MEM software [12]. Subsequently, RNA editing sites were checked by calling SNPs using GATK and comparing to the genomic SNP data [13]. The RNA reads were mapped against their respective chloroplast genome sequence using blastN version 2.2.28 to identify single reads that spanned more than one gene to identify gene clusters that are co-transcribed.

### 2.8 Phylogenetic analysis

The chloroplast genomes of *L. acutangula* and *L. aegyptiaca*, together with 13 chloroplast genomes in the lineage of the Cucurbitaceae family were selected to analyze phylogenetic relationships. The 13 other species were *Cucumis melo* (NC_015983), *Cucumis sativus* (NC_007144), *Coccinia grandis* (NC_031834), *Citrullus lanatus* (NC_032008), *Lagenaria siceraria* (NC_036808), *Cucurbita maxima* (NC_036505), *Cucurbita moschata* (NC_036506), *Cucurbita pepo* (NC_038229), *Trichosanthes kirilowii* (NC_041088), *Hodgsonia macrocarpa* (NC_039628), *Momordica charantia* (NC_036807), *Siraitia grosvenorii* (NC_043881), and *Gynostemma pentaphyllum* (NC_029484). *Oryza sativa* (NC_031333) and *Arabidopsis thaliana* (NC_000932) were also included as outgroups. Sixty-six protein coding genes, conserved among these 17 species (Table S3), were aligned using Kalign software [14], and a phylogenetic tree was constructed using MEGA-X software [15] with the maximum likelihood (ML) method. Bootstrap analysis was calculated by 1000 replications for correction.

## Declaration of Competing Interest

The authors declare that they have no known competing financial interests or personal relationships which have, or could be perceived to have, influenced the work reported in this article.

## Appendix Supplementary Materials

Image, application 1
